# Outcomes of the One Anastomosis Gastric Bypass with Various Biliopancreatic Limb Lengths: a Retrospective Single-Center Cohort Study

**DOI:** 10.1007/s11695-021-05555-y

**Published:** 2021-07-20

**Authors:** Nienke Slagter, Loek J. M. de Heide, Ewoud H. Jutte, Mirjam A. Kaijser, Stefan L. Damen, André P. van Beek, Marloes Emous

**Affiliations:** 1 Center for Obesity Northern-Netherlands (CON), Department of Bariatric and Metabolic Surgery, Medical Center Leeuwarden, Leeuwarden, the Netherlands; 2 Department of Endocrinology, University of Groningen, University Medical Center Groningen, Groningen, the Netherlands

**Keywords:** Bariatric surgery, One anastomosis gastric bypass, Mini gastric bypass, Biliopancreatic limb length

## Abstract

**Introduction:**

One anastomosis gastric bypass (OAGB) is an effective and safe treatment for morbidly obese patients. Longer biliopancreatic (BP) limb length is suggested to result in better weight loss outcomes, but to date, no data are available for the OAGB to substantiate this. We hypothesized that applying a longer BP-limb length in the higher BMI classes would result in more weight reduction so that the attained BMI would be comparable to patients with a lower BMI, thereby compensating for differences in baseline BMI.

**Method:**

A retrospective cohort study in patients who underwent a primary OAGB at a teaching hospital in the Netherlands between January 2015 and December 2016. BP-limb length was tailored based on preoperative BMI. Patients were divided into three different groups depending on the length of the BP-limb: 150, 180, and 200 cm. Weight loss outcomes after 1 and 3 years and resolution of comorbidities were compared between these groups.

**Results:**

Of the 632 included patients, a BP-limb length of 150 cm was used in 172 (27.2%), 180 cm in 388 (61.4%), and 200 cm in 72 (11.4%) patients. Despite more BMI loss, %EWL was lower and attained BMI remained higher in the groups with longer BP-limb lengths. After adjustment for the confounder preoperative BMI, longer BP-limb lengths were not associated with higher BMI loss. There was no difference in remission rates of comorbidities.

**Conclusion:**

Attained BMI remained higher in spite of tailoring BP-limb length according to baseline BMI with no differences in remission rates of comorbidities.

**Graphical Abstract:**

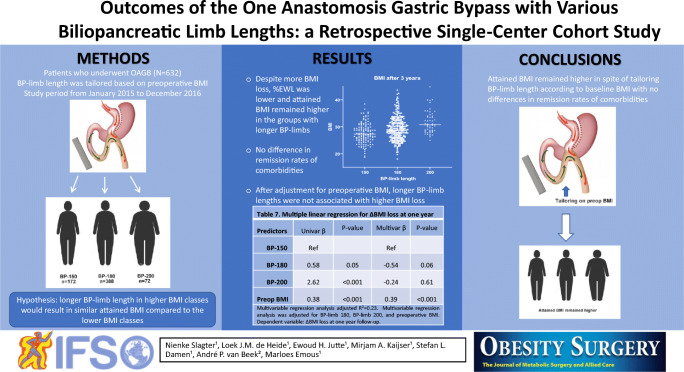

## Introduction

The Roux-en-Y gastric bypass (RYGB) is considered the gold standard procedure in bariatric surgery for the treatment of morbid obesity [1]. There is still no consensus about the optimal lengths of the alimentary and biliopancreatic (BP) limbs in the RYGB. However, some direction to this discussion is given by studies with different limbs lengths to achieve optimal results in terms of weight loss while minimizing the chance of nutritional deficiencies. These studies show that a longer alimentary limb has no effect on weight loss outcomes [2–4]. In contrast, longer BP-limbs result in better weight loss overall [5].

In 1997, Rutledge introduced the one anastomosis gastric bypass (OAGB). Compared to the RYGB, the OAGB has one anastomosis instead of two, which translates into technical ease, shorter operation time, and lower complication rates [6, 7]. Furthermore, the OAGB is easier to revise or reverse and shows equivalent results in weight loss and comorbidity resolution [6–8]. Similar to the RYGB, there is no standard guideline for the optimal BP-limb length in the OAGB. The effect of the BP-limb length in the OAGB is studied to a much lesser extent compared to the RYGB. There is substantial variation in BP-limb length used among bariatric surgeons in the OAGB. Some use a fixed length ranging from 150 to 250 cm and some tailor the BP-limb based on patient-related parameters such as initial body mass index (BMI), age, sex, diet, and comorbidities [6–12]. These subjective variations limit the possibility to compare studies in literature, and therefore hinder consensus on the optimal BP-limb length.

Several years ago, we made it practice to adjust BP-limb length based on preoperative BMI in our hospital. Our hypothesis was that a longer BP-limb in patients with higher BMI would result in more weight loss, so that the final attained BMI would be similar compared to patients with a lower preoperative BMI. The current study aimed to investigate the impact of various BP-limb lengths based on preoperative BMI in terms of weight loss and resolution of comorbidities.

## Methods

### Study Population

This is a single-center retrospective cohort study of all patients who underwent a primary OAGB in a non-academic teaching hospital in the Northern Netherlands, from January 2015 to December 2016 (N = 744). Exclusion criteria were lost to follow-up (n = 29), pregnancy (n = 12), BMI > 50 kg/m^2^ (n = 58), unknown BP-limb length (n = 2), and a BP-limb length of 250 cm (n = 11). All data were extracted from the electronic patient records. The medical ethical committee approved the study (RTPO Leeuwarden, nWMO 20200036). All patients who underwent bariatric surgery had an operation indication in agreement with the international IFSO guidelines and provided written informed consent for the use of their data.

### Preoperative Work-up

Preoperative workup included multiple months of counseling provided by a dietician to prepare for the postoperative lifestyle regimen, including eating 6–8 times a day, separating eating and drinking, eating high-protein and healthy nutritional products, sufficient physical activity, and abstinence of alcohol and carbonated drinks. Candidates were screened by a multidisciplinary team evaluating mental health, detailed medical condition, and adherence to the postoperative lifestyle.

### Surgical Technique

The surgical technique has been described before by Apers et al. [13]. The BP-limb was measured from Treitz’ ligament. The jejunum was brought up antecolic-antegastric, and the gastrojejunostomy was created using an endoscopic linear stapler loaded with a 60-mm cartridge (Ethicon Echelon Flex Powered). The gastrojejunostomy was closed manually using an absorbable suture. Application of an anti-reflux suture, attaching the proximal jejunum at the left lateral side of the gastric pouch using one suture, was based on surgeon’s preference [14]. A BP-limb length of 150, 180, or 200 cm was used. As a general rule, a 150-cm BP-limb was used for BMI < 40 kg/m^2^, a 180-cm BP-limb for BMI 40–44.9 kg/m^2^, and a 200-cm BP-limb for BMI 45–49.9 kg/m^2^. The ultimate BP-limb length was determined per-operative at the discretion of the surgeon and therefore could differ from the general rule. The small bowel was measured with a hand-over-hand technique, with 5 cm steps for measuring purposes. The standard instrument had a 4.5-cm metallic grasper and was used as a reference. All OAGB procedures were performed either by four experienced bariatric surgeons or by surgical residents under direct supervision of these bariatric surgeons.

### Follow-up

Weight loss surgery specific multivitamins and calcium/vitamin D supplementation were recommended lifelong. The patient’s follow-up visits took place after 1 month, 1 year (10–14 months), 2 years (18–30 months), and 3 years (30–42 months). Follow-up data were included until the 1st of June 2020. At each follow-up visit, standard care included inquiry after well-being and complaints, current medication use, side effects, evaluation of comorbidities, and measurement of body weight.

The percent of excess weight loss (%EWL) was defined as (initial weight– postoperative weight) / (initial weight – ideal weight) x 100. Ideal weight was defined as the weight corresponding to a BMI of 25 kg/m^2^. The percent of total weight loss (%TWL) was defined as (initial weight – postoperative weight) / initial weight x 100. Remission of hypertension and diabetes mellitus type 2 (T2D) was defined as partial if the dosage of medication was decreased compared to the preoperative dosage. Total remission of hypertension and T2D was defined as discontinuation of the prescribed medication with normalization of the blood pressure or HbA1c.

### Statistics

Data were presented as mean ± standard deviation, median [interquartile range], and number (percentages). Normally distributed values were compared using a t-test or one-way ANOVA, skewedly distributed variables were compared using the Mann-Whitney U test or Kruskal-Wallis test, and for categorical data, the Chi-squared test was used. A two-sided p-value of ≤ 0.05 was considered statistically significant. Bonferroni correction was applied when post hoc tests were performed.

Multiple linear regression analyses were performed to analyze the effect of the different BP-limb lengths on %EWL and BMI loss when correcting for confounders. In both multiple regression analysis, preoperative BMI was selected as confounder. Statistical analyses were performed in SPSS version 24.

## Results

Baseline characteristics of the 632 patients are shown in Table 1. The mean age was 48 ± 11 years, and 525 patients were female (83%). Mean preoperative weight and BMI were 124 ± 17 kg and 42 ± 4 kg/m^2^, respectively. Hypertension was documented in 209 (33%) and T2D in 126 (20%) patients. Late postoperative complications as ulcers were present in 3.6%, internal herniation in 0.6%, and cholelithiasis in 5% of the patients. Two patients died during the follow-up, not related to the OAGB procedure (0.3%). Follow-up percentages after 1, 2, and 3 years were 94, 86, and 74%, respectively.

**Table 1:** Baseline characteristics

Variables	N = 632
Female	525 (83%)
Age, years	48 ± 11
Preoperative weight, kg	124 ± 17
Height, cm	172 ± 8
BMI, kg/m^2^	42 ± 4
Hypertension	209 (33%)
T2D	126 (20%)
BP-limb length, cm	
150	172 (27%)
180	388 (61%)
200	72 (11%)

Values are mean ± standard deviation, or number (%) of subjects

*BMI*, body mass index; *T2D*, diabetes mellitus type 2; *BP*, biliopancreatic

### Weight Loss and Remission of Comorbidities in the Total Group

The mean BMI loss after 1 and 3 years was 13 ± 3 and 13 ± 4, respectively, resulting in a BMI of 29 ± 4 kg/m^2^ in both years (Table 2). Mean %EWL after 1 year was 80 ± 20 % and 77 ± 24 % after 3 years. One and three years postoperative %TWL was 31 ± 7 % and 30 ± 9 %, respectively. Total resolution of hypertension was seen in 107 (51%) patients and partial remission in 82 (39%) patients (Table 3). Resolution of T2D was achieved in 93 (74%) patients and improved in 31 (25%).

**Table 2:** Weight loss

Variables	1 year	2 years	3 years
N	596	543	465
BMI, kg/m^2^	29 ± 4	28 ± 4	29 ± 4
∆ BMI, kg/m^2^	13 ± 3	14 ± 4	13 ± 4
WL, kg	39 ± 10	41 ± 13	37 ± 13
%EWL	80 ± 20	84 ± 23	77 ± 24
%TWL	31 ± 7	33 ± 9	30 ± 9

Values are mean ± standard deviation

*BMI*, body mass index; *WL*, weight loss; *EWL*, excess weight loss; *TWL*, total weight loss

**Table 3:** Resolution of comorbidities

Comorbidity	Hypertension	T2D
N (preoperative)	209	126
Total remission	107 (51%)	93 (74%)
Partial remission	82 (39%)	31 (25%)
No remission	19 (9%)	0 (0%)
Unknown	1 (1%)	2 (1%)

Values are number (%) of subjects

*T2D*, diabetes mellitus

### Weight Loss and Comorbidities in Different BP-Limb Lengths

**Fig. 1:**
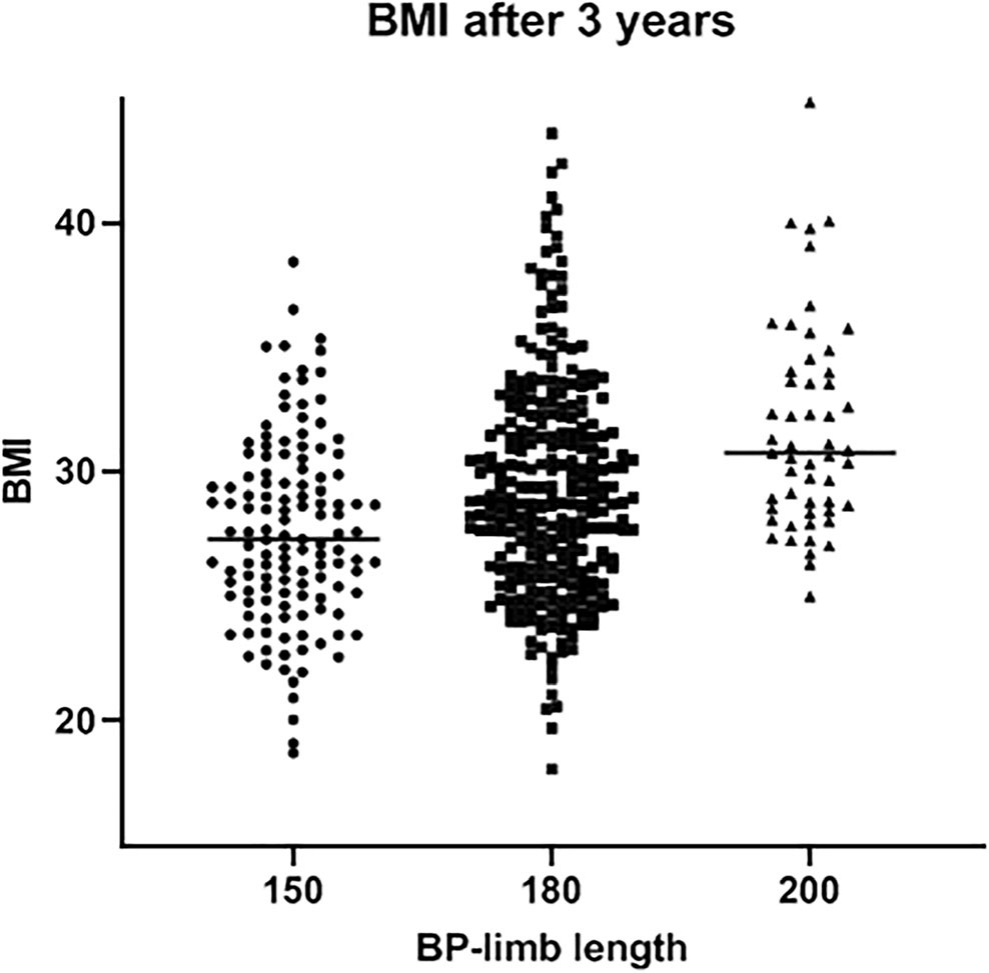
Attained BMI after 3 years for the different lengths of the BP-limb

Patients were categorized into three groups based on the length of the BP-limb: 150 cm in 172 (27.2%), 180 cm in 388 (61.4%), and 200 cm in 72 (11.4%) patients (Table 4). There was no difference in sex and age between the three groups (P = 0.07 and P = 0.47). As expected, preoperative BMI was higher in the groups with longer BP-limb lengths. Postoperative weight loss results for the different groups are shown in Table 4 and Fig. 1.

**Table 4:** Weight loss for different BP-limb lengths

	150	180	200	P-value
N = 632	N = 172	N = 388	N = 72	
Female	146 (85%)	326 (84%)	53 (74%)	0.07
Age, years	48 ± 10	49 ± 11	47 ± 11	0.47
Preoperative weight, kg	113 [104–126]	122 [114–133]^1^	141 [128–151]^1 2^	< 0.001
Preoperative BMI, kg/m^2^	39 [37–41]	42 [40–45]^1^	47 [44–48]^1 2^	< 0.001
Year 1	N = 156	N = 374	N = 66	
BMI, kg/m^2^	26 [25–29]	29 [27–32]^1^	31 [29–34]^1 2^	< 0.001
∆ BMI, kg/m^2^	12 [10–15]	13 [11–15]	16 [13–17]^1 2^	< 0.001
WL, kg	36 [30–43]	37 [33–44]	48 [39–53]^1 2^	< 0.001
%EWL	89 [74–103]	78 [65–90]^1^	73 [61–83]^1 2^	< 0.001
%TWL	32 [27–36]	31 [27–35]	33 [29–37]^2^	0.035
Year 3	N = 129	N = 283	N = 53	
BMI, kg/m^2^	27 [25–30]	29 [26–32]^1^	31 [28–34]^1 2^	< 0.001
∆ BMI, kg/m^2^	11 [9–15]	13 [10–16]^1^	15 [12–16]^1 2^	< 0.001
WL, kg	33 [25–42]	38 [29–45]^1^	47 [36–56]^1 2^	< 0.001
%EWL	83 [65–99]	77 [61–93]^1^	75 [59–81]^1^	0.002
%TWL	29 [23–36]	31 [25–37]	34 [28–38]	0.148

Values are mean ± standard deviation; median [interquartile range]; or number (%) of subjects

*BP*, biliopancreatic; *BMI*, body mass index; *WL*, weight loss; *EWL*, excess weight loss; *TWL*, total weight loss

^1^Significant difference compared to the BP-limb 150 group. ^2^ Significant difference compared to the BP-limb 180 group

After 1 postoperative year, attained BMI was significantly higher in the groups with longer BP-limbs. The BP-200 cm group had more BMI loss and WL compared to the 150 and 180 cm groups with no significant difference between the latter two. The %EWL was significantly higher in groups with shorter BP-limb lengths. The %TWL was higher in the group with a BP-limb of 200 cm compared to the group with a BP-limb of 180 cm (33%, 31%, P = 0.01), the other groups showed no difference.

After 3 years, attained BMI, BMI loss, and WL were significantly higher in groups with longer BP-limbs. %EWL was significantly higher in the 150-cm group compared to the other two groups, with no difference between patients in the groups of 180 and 200 cm. No difference in %TWL between the groups was found. As shown in Table 5, there was no significant difference in remission of hypertension and T2D between the three groups.

**Table 5:** Resolutions of comorbidities and revision surgery for different BP-limb lengths

Variables	150	180	200	P-value
N = 632	N = 172	N = 388	N = 72	
Hypertension (preoperative)	58 (34%)	130 (34%)	21 (29%)	0.76
Remission hypertension				
Total	28 (49%)	69 (53%)	10 (48%)	0.83
Partial	24 (42%)	47 (36%)	11 (52%)	0.33
None	5 (9%)	14 (11%)	0 (0%)	0.28
T2D (preoperative)	31 (18%)	80 (21%)	15 (21%)	0.76
Remission T2D				
Total	21 (68%)	59 (76%)	13 (87%)	0.37
Partial	10 (32%)	19 (24%)	2 (13%)	0.37
Conversion to RYGB	22 (13%)	38 (10%)	9 (13%)	0.52
Revision BP-limb	2 (1%)	1 (0.3%)	0 (0%)	0.29
Undo	1 (1%)	0 (0%)	0 (0%)	0.25
Suboptimal weight loss at one year follow-up				
%EWL < 50	4 (2%)	26 (7%)	6 (8%)	0.08

Values are number (%) of subjects

*T2D*, diabetes mellitus type 2; *RYGB*, Roux-en-Y gastric bypass; *BP*, biliopancreatic; *BMI*, body mass index; *EWL*, excess weight loss

### Multiple Linear Regression Analyses

After adjustment for preoperative BMI, a BP-limb length of 180 cm was significantly associated with 4,5% less %EWL at 1 year follow-up, compared to a BP-limb of 150 cm (P = 0.01) (Table 6). After adjustment for the same variable, a BP-limb length of 200 cm had no significant effect on %EWL after 1 year follow-up (β = − 0.6, P = 0.84). Different BP-limb lengths had no significant effect on BMI loss after 1 year follow-up, after controlling for the confounder preoperative BMI (Table 7).

**Table 6:** Multiple linear regression for %EWL at 1 year follow-up

Predictors	Univariate β	P-value	Multivariable β	P-value
BP-limb length, cm				
150	Ref		Ref	
180	− 11.6	< 0.001	− 4.5	0.01
200	− 18.9	< 0.001	− 0.6	0.84
Preoperative BMI, kg/m^2^	− 2.6	< 0.001	− 2.5	< 0.001

Multivariable regression analysis adjusted R^2^ = 0.27. Multivariable regression analysis was adjusted for BP-limb 180, BP-limb 200, and preoperative BMI. Dependent variable: %EWL at 1 year follow-up

**Table 7:** Multiple linear regression for ∆BMI loss at 1 year follow-up

Predictors	Univariate β	P-value	Multivariable β	P-value
BP-limb length, cm				
150	Ref		Ref	
180	0.58	0.05	− 0.54	0.06
200	2.62	< 0.001	− 0.24	0.61
Preoperative BMI, kg/m^2^	0.38	< 0.001	0.39	< 0.001

Multivariable regression analysis adjusted R^2^ = 0.23. Multivariable regression analysis was adjusted for BP-limb 180, BP-limb 200, and preoperative BMI. Dependent variable: ∆BMI loss at 1 year follow-up

## Discussion

The results of this retrospective cohort study show that longer BP-limbs in patients with a preoperative higher BMI did not result in an attained BMI that was similar to the patients with a preoperative lower BMI. Furthermore, longer BP-limb lengths were not associated with higher BMI loss, after adjustment for the confounder preoperative BMI.

Patients were selected for different BP-limb length based on their preoperative BMI. Our hypothesis was that a longer BP-limb in patients with higher BMI would result in more weight loss, so that final BMI in those patients would be similar to those with a preoperative lower BMI. The results of this retrospective cohort study do not support this hypothesis. The more BMI loss in the groups with the longer BP-limbs seems to be mainly caused by higher preoperative BMI and not by the longer length of the BP-limbs. This effect of preoperative BMI on weight loss outcomes corresponds with a retrospective cohort study on predictors of weight loss after RYGB in 2070 patients [15]. Higher preoperative BMI also resulted in a higher postoperative BMI and lower %EWL. Furthermore, we found no difference in remission rates of T2D or hypertension between the groups with different BP-limb lengths.

Using the percentage %EWL or %TWL as outcome when comparing different BMI groups introduces a bias [16]. A higher initial weight results in a higher number in the denominator of the fraction. As shown in our results, even with more weight loss, the percentage %EWL is still lower in the preoperative heavier patients due to the higher initial weight. Therefore, the absolute measurements of weight or attained BMI are more informative than %EWL and %TWL.

Retrospective cohort studies on tailoring BP-limb based on BMI in OAGB show comparable results in terms of weight loss outcomes [17–19]. These studies found higher BMI and lower %EWL, despite more BMI loss and weight loss in the groups with both a longer BP-limb and higher preoperative BMI. Kermansaravi et al. analyzed the impact of the BP-limb length, adjusted based on preoperative BMI and age in 653 patients who underwent an OAGB [19]. They found that preoperative weight was one of the most contributory predictors and BP-limb length the least contributory predictor of %EWL. Furthermore, they found no difference in comorbidities remission rates between the different limb lengths. Due to the higher weight and BMI loss, the authors of these retrospective studies conclude that tailoring the BP-limb based on preoperative BMI demonstrated satisfying results and they recommend this strategy. Results from our study suggest that the higher BMI loss in patients with longer BP-limb lengths is most likely predominantly caused by the higher preoperative BMI and not by the longer BP-limb lengths themselves.

The literature on the OAGB contains considerable variation in lengths used for the BP-limb. Some bariatric surgeons use a fixed length and some tailor the BP-limb based on patient-related parameters such as BMI, age, sex, diet, and comorbidities [6, 8–12, 17, 20–22]. The small number of retrospective studies comparing different BP-limb lengths contain subjective variations, which limit the possibility to compare the studies. This lack of literature hinders obtaining consensus on the best way to determine the BP-limb length in the OAGB. The effect of variable limb lengths in RYGB has been studied more extensively compared to the OAGB. For both the alimentary and BP-limb, there is no consensus about the optimal length. Several studies showed no difference in weight loss outcomes for variable lengths of the alimentary limb [2–4]. Nergaard et al. performed an RCT comparing a RYGB with a BP-limb of 200 to 60 cm and an alimentary limb of 60 and 150 cm, respectively. The longer BP-limb length led to more weight loss but also to more bowel movements and micronutrient deficiencies [23]. Furthermore, Zorrilla et al. performed a systematic review on limb length in RYGB and found 13 studies meeting adequate quality [5]. Weight loss on the whole was better in patients with longer BP-limbs.

The length of the total small bowel can vary considerably, with measured values ranging from 350 to 1050 cm [24]. The effect of a BP-limb of 150 cm may be different for an obese patient with a total small bowel of 400 cm, compared to a patient with a total small bowel of 800 cm. Two recent retrospective studies showed promising results of more weight loss and less nutritional deficiencies when tailoring the BP-limb based on the total small bowel length [25, 26]. A study of Tacchino et al. identified predictors of the total small bowel length and found no correlation between weight and small bowel length [24]. In our study, the total small bowel length or the length of the common channel was not measured. This might explain why the length of the BP-limb does not seem to influence the BMI loss in our study population.

In our study population, satisfactory results of mean weight loss outcomes were seen. After 3 years of follow-up, BMI and %EWL were 29 kg/m^2^ and 77%, respectively. These results are similar to other large series reported by bariatric centers who perform the OAGB [6, 8–10, 21]. When evaluating studies on weight loss results, not only the average but also the distribution is important. There is a wide distribution in weight loss outcomes in our study population as shown in Fig. 1. Mean BMI after 3 years was 29 ± 4 kg/m^2^ and %EWL 77 ± 24 %. This wide distribution of outcomes in BMI and %EWL are comparable with other large cohort studies in both OAGB and RYGB [27, 28]. Weight loss surgery aims to achieve an optimum weight loss striving for a metabolically healthy BMI of 25 kg/m^2^ in combination with a minimum of side effects. After 3 years of follow-up, 91% of the patients still had a BMI above 25 kg/m^2^. With the aims in mind, there is clearly room for improvement in bariatric procedures. Remission of hypertension and T2D both total and partial occurred in 90% and 99% of the patients, respectively. These results are comparable to other large cohort studies on OAGB [6, 10, 12, 13, 21].

This study emphasizes that further research is necessary to find the optimal way to determine the BP-limb length in the OAGB. The contribution of the total length of the small bowel to weight loss and nutritional deficiencies in OAGB needs further exploration. We have therefore started a randomized controlled trial comparing a fixed BP-limb length to one tailored based on total small bowel length looking at both achieving a healthy BMI with narrow range and few nutrient deficiencies (TAILOR study, ISRCTN 00001082).

The strength of the present study lies in the large and representative population, with a relatively large number of participants in each group. The lost to follow-up over 3 years was 26%, which are realistic data from a bariatric center in the Netherlands. Limitations of this study were the retrospective study design and the lack of data on nutritional deficiencies and total small bowel length. As some studies found longer BP-limb lengths were associated with more nutritional deficiencies, this would have been a valuable addition to this study [20].

In conclusion, in this retrospective study, tailoring BP-limb length according to baseline BMI did not abrogate the BMI differences at 3 years after OAGB with also no differences in resolution of comorbidities.
